# Prevalence of non-communicable diseases and their risk factors at a semi-urban community, Pakistan

**DOI:** 10.11604/pamj.2016.23.151.8974

**Published:** 2016-03-31

**Authors:** Sajida Naseem, Umme Kulsoom Khattak, Haider Ghazanfar, Awais Irfan

**Affiliations:** 1 Shifa College of Medicine, Shifatameer E Millat University, Islamabad, Pakistan

**Keywords:** Non-communicable diseases, epidemiology, Pakistan, life style diseases, health

## Abstract

**Introduction:**

Pakistan is currently facing the double burden of communicable (38%) and non- communicable diseases (49%) according to WHO NCD Country Profiles 2014. About 50% of all deaths are attributed to NCD's. The objective of this study was to determine the burden of non-communicable diseases in semi urban community of Islamabad.

**Methods:**

We carried a cross sectional study to estimate the burden of non-communicable diseases in an urban setting, a community based cross sectional survey covering 1210 households was carried out over a period of three months. Households were selected through consecutive non-probability sampling, among which adult females and males who were permanent resident of the community were interviewed through a structured questionnaire in urdu language. SPSS version 21 was used to analyze the data. Descriptive statistics were calculated.

**Results:**

About 38.7% individuals had High BP / IHD, 34.4% had oro-dental health problems, 24.3% were physically disabled and 14.6% had diabetes. Among the risk factors, 48.2% were tobacco user, 13.60% were drug abuser and 1.8% alcoholics.

**Conclusion:**

We conclude that the prevalence of non-communicable diseases is quite high in the above setting as compared to the National indicators, which demands timely intervention to curtail the existing burden of NCD.

## Introduction

About 38 million people die every year due to non-communicable disease [1]. Out of this 70-80% deaths occur in low and middle-income countries [1–3]. About 18% of global deaths are attributed to elevated blood pressure [4]. Over the last decade the NCDs burden has increase resulting in a barrier to development goals including poverty reduction, human security, economic stability and health equity [5]. The increase in NCD can be due to increasing longevity and due to societal & cultural changes such as increasing tobacco and illicit drug use [6]. Pakistan is currently facing the double Burden of Communicable (38%) and Non- Communicable Diseases (50%) according to WHO NCD Country Profiles 2014. The WHO country profile (2014) shows that in Pakistan 25.3% individuals had high BP,19% had CVD diseases, 3% had diabetes, 6% had chronic respiratory diseases, 8% had cancers, 23% were tobacco smokers and 0.1% were alcohol consumers [7]. The objective of this study was to determine the burden of non-communicable diseases in semi urban community of Islamabad.

## Methods

We performed a community based cross sectional survey using a self-made questionnaire covering 1210 households in Nurpur Shahan over a period of 3 months from Dec 2014 to February 2015. Households were selected through consecutive non-probability sampling, among which adult males and females were interviewed through a structured questionnaire in Urdu language. Using WHO sample size calculator, keeping 95% confidence level, and prevalence of DM 3%, absolute precision required 1%; sample size of 1200 was calculated. Only permanent residents of Nurpur Shahan were included in this study. Residents who were less than 18 years of age were not interviewed. Informed consent was obtained from all the participants. SPSS version 21 was used to analyze the data. Mean and Standard deviation was calculated for all quantitative variables. Frequency and percentages was calculated for qualitative variable. Pearson's chi square test was used to find association of tobacco use, drug abuse and alcohol with Non-communicable disease. A p value of less than 0.05 was considered significant. Ethics approval for this study was obtained from Shifa International Hospital review board and ethical committee.

## Results

**Figure 1:**
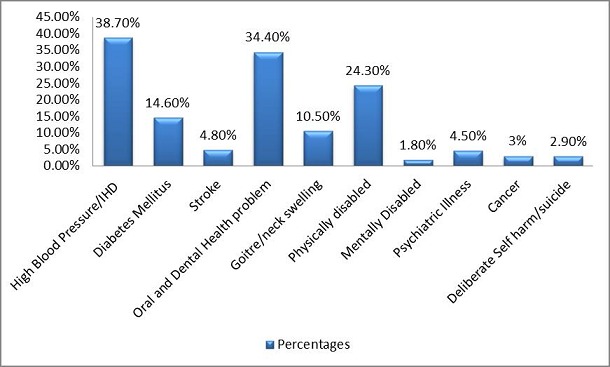
Frequency of non-communicable diseases

**Figure 2:**
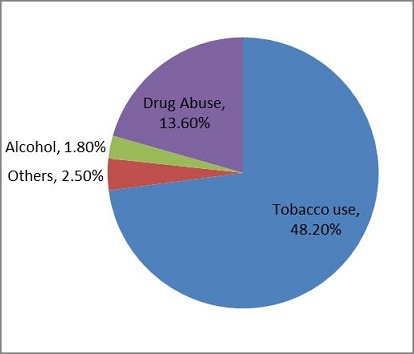
Risk factors for non-communicable disease

The mean age of the participant was 31.41 (SD 8.256) and the mean number of family member were 7.18 (SD 3.41). Mean number of earning family member was 1.51 (SD 0.93) and the mean month family income was 643 Pak Rupees (SD 3078.68). Frequency of Non-communicable diseases has been shown in Figure 1. In our study tobacco use was present in 583 (48.2%) household, drug abuse in 164 (13.6%) households and alcohol use in 22 (1.8%). This has been shown in Figure 2. Alcohol intake was found to be significantly (p < 0.05)associated with stroke, oral and dental health problem, committed deliberate self-harm/suicidal ideation. Drug Abuse was found to be significant (p < 0.05) associated with high blood pressure/Ischemic Heart Disease, oral and dental health problem committed deliberate self-harm/suicidal ideationand Psychiatric illness.

## Discussion

While the sample size of this study clearly does not include the entire population of Pakistan, it is a fairly accurate reflection of semi-urban community. Keeping this in mind, it is worthwhile to note that such drastic increases in frequencies of patients who smoke, drink alcohol, and have diabetes, may have happened within a small time frame; emphasizing the need for interventional programs. In this study, the biggest non-communicable disease risk factor we face is hypertension. Lloyd-Sherlock P et al performed a study in certain low to middle income countries, and also found very a very high prevalence of hypertension in these countries [8]. The aforementioned study also examined the levels of awareness each country's population had with regard to their condition; approximately 50% of people were aware, but few actually had their blood pressure under control according to the authors, thus highlighting not only an awareness problem, but also the urgent need to educate patients about the serious nature of this seemingly benign diagnosis, and how to treat it. Our study also investigated the relation of hypertension to various stress factors, for example a low monthly income. Mushtaq and Najam conducted a study which linked hypertension to psychiatric illness and stress, and found a strong positive correlation [9]. Among the stress variables outlined in the study, monthly income, number of dependents, and psychiatric illnesses like depression weighed in as the major factors contributing to hypertension. Meng L et al also tried to correlate depression, a type of psychiatric illness, to hypertension. They found that depression was indeed a risk factor for hypertension [10]. Our findings support the above. In our study, of the 464 households that reported someone in their family having high blood pressure, the majority fell under the category of relatively low income (148 had a monthly income of 5,000-10,000, and 108 between 10,000-15,000) and the general trend was that the higher the monthly income, the less the prevalence of high blood pressure. However, our research showed that psychiatric illness does not correlate well with hypertension. Of the 54 households that reported someone in their house with psychiatric illness, only 24 households also had someone with high blood pressure, as opposed to 30 with no high blood pressure. Knowing that hypertension is one of the leading causes of morbidity and mortality worldwide, tackling this is a major area of concern. The treatment of high blood pressure is impeded by a number of barriers, outlined by Khatib R et al in a meta-analysis. They found that the most common reason for lack of control was lack of knowledge of risk factors like smoking, but stress and anxiety played a role in the inability to control high blood pressure [11]. Oral hygiene and General Health go side by side. Dental Caries are the most common child hood non-communicable disease in childhood [12]. Disability-adjusted life years (DALYs) due to oral condition have increased by 20.8% between 1990 and 2010 [13]. Helen Clark has stated that “focus on oral health in overall primary health care will not only help improve oral health itself, but will also reduce the rate of cardiovascular diseases, cancer, chronic respiratory diseases, and diabetes” [14]. In our study Oral and Dental problem was the second most common non-communicable disease. In a study done in Nigeria about one-third of the participant has dental caries [15]. The fact which is even more alarming is that most Pediatricians lack proper awareness of dental caries [16–17]. There is a dire need toformulate strategies to nip this issue in the bud.

Analysis of physical disability revealed some fascinating results. The highest frequency of physically disabled was in the age of 35 (44), followed by ages 25 (24) and 30 (22) respectively. It was expected that the elderly would be more disabled than the young adults, however that is not the case as displayed by the data. The type of physical disability present in the population in this study is not known; however, the most serious, and indeed, the one that has put Pakistan under the spotlight in recent years, is poliomyelitis. Farag NH et al report that in the time period from January 2014 to August 2014, there were 170 cases reported, rising from a number of 93 cases in the whole of 2013, and 58 in the preceding year [18]. It thus becomes necessary to ascertain the type of disability affecting so many young people, and whether it is associated with communicable disease or not. Diabetes Mellitus was found to be the 4th most common Non-communicable disease in our study. Urbanization in South East Asia has resulted in mushrooming of coronary heart disease, diabetes and respiratory diseases [19]. Diabetes is projected to be the 7thleading cause of death by 2030 [20]. Physical inactivity, overweight and obesity have significantly contributed to the increase in global prevalence of diabetes [21]. Diabetes is a well-known risk factor for cardiovascular diseases [22], blindness [23], kidney failure [24], limb amputation and premature death [25]. Cancer is the leading causes of death worldwide. The numbers of new cases of cancers are expected to increase by 70% over the next 2 decades [26]. This increase is expected to be more in low and lower middle income countries as compared to upper middle and upper income countries. Tobacco use, alcohol use, unhealthy diet and physical inactivity are the main cancer risk factors. About 40% cancer can be avoided by regular physical activity, healthy diet and avoidance of tobacco-use [27]. With proper awareness and education the prevalence of the cancer can be significantly reduced. Tobacco use and drug addiction is a rising menace and is consuming our society at an alarming rate. In our study tobacco use was found in 48.2% household. Results were similar to a study done in Delhi [28]. Tobacco use is more prevalent in rural areas as compared to urban areas [28–29]. Tobacco use is also a risk factor for communicable diseases [30]. If remained unchecked deaths related to it are projected to increase to 8 million by 2030 which accounts for 10% of all death. At least 15.3 million people have drug use disorders and harmful use of alcohol results in 3.3 million deaths each year [31]. Awareness campaigns about the deleterious effects of Tobacco, drug abuse and alcohol use are the need of the hour. Volunteer members of the community can be selected and trained to lead such campaigns. This will increase the chance of community participation in such programs [32]. Along with this screening and treatment should be provided at school level to prevent and decrease the frequency of Alcohol and other drug use among children and adolescents [33]. Our study shows an alarming rate of deliberate self-harm and suicide rate. Correlating this with psychiatric illness, we can see that the majority of households reporting family members who have committed suicide or attempted some form of self-harm, in fact, do not have any previous history of psychiatric illness (p > 0.05). Evidence suggests that there are other factors involved that would lead the population to commit acts of self-harm, for example unfavorable home conditions, unemployment [34], female gender [35], age [36] and physical abuse. Having a suicidal past was found to be associated with both attitudinal and stigmatizing barriers towards helps seeking and accepting attitudes towards suicide [37]. Efforts can be made in future studies to identify various factors leading to suicide in different communities and thereafter begin to do away with them. Devising, and even more so, implementing, strategies to cope with the burden of non-communicable diseases, is proving to be fairly difficult. Lack of television, internet, and even radio, in the vast majority of peripheral regions of Pakistan makes media campaigns non-feasible. In Pakistan, local health workers (especially LHW's) are a well-established concept, and therefore a more viable option would be to educate the LHW's station in each village, and provide them with the responsibility of employing the appropriate healthcare guidelines. To overcome these barriers, we must find effective large scale strategies to educate the population and eliminate risk factors in order to gain some control over the burgeoning burden of non-communicable diseases. This is especially important for low and middle income countries, which are now currently experiencing double burden due to communicable diseases and non-communicable diseases.

## Conclusion

We conclude that the prevalence of Non-Communicable diseases is quite high in the above setting as compared to the National indicators, which demands timely intervention to curtail the existing burden of NCD's. Also there is a need to update the statistics at least the regional level. There is a need for Health Awareness and Health education strategies regarding Non Communicable diseases and their risk factors.

### What is known about this topic

- About 38 million people die every year due to non-communicable disease. Out of this 70-80% deaths occur in low and middle-income countries.
- Over the last decade the NCDs burden has increase resulting in a barrier to development goals including poverty reduction, human security, economic stability and health equity.
- Pakistan is currently facing the double Burden of Communicable (38%) and Non- Communicable Diseases (50%) according to WHO NCD Country Profiles 2014.

### What this study adds

- We conclude that the prevalence of Non-Communicable diseases is quite high in the above setting as compared to the National indicators.
- There is a need to educate the LHW's station in each village, and provide them with the responsibility of employing the appropriate healthcare guidelines.
- Awareness campaigns about the deleterious effects of Tobacco, drug abuse and alcohol use are the need of the hour.
